# The Survival Benefit for Optimal Glycemic Control in Advanced Non-Small Cell Lung Cancer Patients With Preexisting Diabetes Mellitus 

**DOI:** 10.3389/fonc.2021.745150

**Published:** 2021-11-16

**Authors:** Jie Qian, Weimin Wang, Lin Wang, Jun Lu, Lele Zhang, Bo Zhang, Shuyuan Wang, Wei Nie, Yanwei Zhang, Yuqing Lou, Baohui Han

**Affiliations:** ^1^ Department of Emergency Medicine, Shanghai Chest Hospital, Shanghai Jiao Tong University, Shanghai, China; ^2^ Department of Pulmonary Medicine, Shanghai Chest Hospital, Shanghai Jiao Tong University, Shanghai, China; ^3^ Department of Pulmonary Function, Shanghai Chest Hospital, Shanghai Jiao Tong University, Shanghai, China; ^4^ Department of Laboratory Medicine, Shanghai Chest Hospital, Shanghai Jiao Tong University, Shanghai, China

**Keywords:** non-small cell lung cancer, diabetes, prognosis, glycemic control, glycated hemoglobin A1C (HbA1C)

## Abstract

**Background:**

Diabetes mellitus (DM) is a frequent comorbidity in patients with cancer. This study aimed to evaluate the prognosis of advanced non-small cell lung cancer (NSCLC) patients with DM and to assess whether an optimal glycemic control improves overall survival (OS).

**Methods:**

A total of 1279 advanced NSCLC patients including 300 (23.5%) with preexisting DM were retrospectively reviewed. The continuous relationship between glycated hemoglobin A1C (HbA1c) level and OS was analyzed by restricted cubic spline (RCS) function. Optimal HbA1c cut-off point was determined using X-tile analysis. Survival was analyzed with the Kaplan–Meier method and compared among groups stratified by diabetes status and HbA1c. Multivariable Cox proportional hazards regression analysis was employed to identify prognostic factors for OS after adjusting for baseline characteristics.

**Results:**

DM and non-DM patients had similar OS (median (95% CI): 22.85 (20.05-26.73) *vs.* 22.22 (20.35-24.76) months, P=0.950). The multivariate Cox regression analyses showed that DM status was not a prognostic factor for OS (HR: 0.952, 95% CI: 0.808-1.122, P=0.559). However, there existed a non-linear but generally positive relationship between the elevated HbA1c level and increased risk of overall mortality. HbA1c > 6.6% was a negative prognostic factor for OS (HR: 1.593, 95% CI: 1.113-2.280, P=0.011). The median OS (95% CI) for nondiabetic patients, DM patients with HbA1c ≤6.6% and those with HbA1c > 6.6% was 22.22 (20.01-24.43), 25.28 (21.79-28.77) and 15.45 (7.57-23.33) months, respectively. Well-controlled DM patients had a comparable crude OS (HR (95% CI): 0.90 (0.76-1.08), P=0.273] compared to nondiabetic patients while patients with HbA1c>6.6% had a worse crude OS than patients without DM (HR (95% CI): 1.70 (1.24-2.34), P=0.001]. The survival benefit of good HbA1c control was prominent in all subgroups.

**Conclusion:**

Impaired glycemic level negatively affects survival for patients with advanced NSCLC while proper glycemic control with HbA1c ≤6.6% improves the OS.

## Introduction

Diabetes mellitus (DM) is one of the most frequent comorbidities in patients with cancer (1). In lung cancer patients, approximately 10-20% of patients have preexisting diabetes and the prevalence is anticipated to increase with the growing epidemic of DM (2–5). Lung cancer and DM are both life-limiting diseases sharing similar risk factors of aging and smoking (4, 6, 7). They may mutually affect the treatment strategy and prognosis (7–9). Non-small cell lung cancer (NSCLC) accounts for the majority of lung cancer. A deeper understanding of outcomes and management for NSCLC coexisting with DM is drawing increasing attention. However, prognostic and biological interactions of NSCLC and DM remain largely unknown.

Diabetic status has complicated biological effects on the pathogenesis of cancer. In one way, diabetes may contribute to the growth, proliferation and invasiveness of cancer *via* metabolic remodeling of cancer-associated signaling and enhanced epithelial-to-mesenchymal transition (EMT) mediated by hyperinsulinemia and hyperglycemia (10, 11). In another way, diabetic microangiopathy renders the vascular basal membrane less digestible by tumor cells, thus impeding tumor spread and metastasis (12–14). With regard to the prognostic impact of DM on lung cancer, previous studies showed conflicting results. Some data concluded that the presence of DM was predictive of a negative outcome for lung cancer patients (2, 3, 15–18). The negative impact of preexisting DM claimed to be more remarkable in postoperative NSCLC than NSCLC receiving non-surgical treatment (18). However, prolonged survival in patients with lung cancer and DM was observed in other studies (19, 20). The latest large-scale Asian cohort revealed that DM was not significantly associated with the risk of mortality in NSCLC (21).

For diabetic patients, glycated hemoglobin A1C (HbA1c) is a reliable measurement of glycemic control (22). A good glycemic control reduces the long-term risk of complications in diabetic patients (23). Well-controlled glycemic level was associated with a better survival in breast, pancreatic or colon cancer (24–26). However, few studies evaluated the prognostic impact of proper glycemic control in NSCLC patients. A recent study focusing on resected NSCLC demonstrated that glycemic control with HbA1c<7% was associated with improved overall survival (OS) (27). The precise relationship between HbA1c and survival in advanced NSCLC has not yet been studied.

In the current study, we attempted to investigate two important but largely unaddressed issues: 1) to evaluate the impact of diabetic status on OS in advanced NSCLC, and 2) to further uncover the relationship between HbA1c level and survival. This study would be the first, to the best of our knowledge, to display an impact profile of HbA1c on the prognosis in advanced NSCLC, which is helpful for the management of advanced NSCLC patients with coexisting DM.

## Methods

### Patient and Data Collection

Medical records of patients admitted between January 2012 and December 2015 in Shanghai Chest Hospital were reviewed. The inclusion criteria were a pathologic diagnosis (including cytology or histology) of primary advanced NSCLC staged according to the 8^th^ TNM edition of the American Joint Committee on Cancer (AJCC) cancer staging manual. Patients without complete records of OS were excluded. The study was approved by the institutional ethics committee of the Shanghai Chest Hospital. All patients were anonymized and written signed informed consent was obtained from each participant. The study protocol conformed to the Helsinki Declaration.

Demographic and clinical data including age at lung cancer diagnosis, gender, smoking history, body mass index (BMI), stage, histology, EGFR mutation status, cardiovascular disease (CVD) and treatment regimen were recorded. CVD was defined as having a history of hypertension, coronary heart disease, heart failure, cerebrovascular disease, peripheral vascular disease, rheumatic or congenital heart disease and cardiomyopathies. OS was defined as the time from LC diagnosis until death from any cause. The follow-up was started from the date of LC diagnosis.

### Diabetes Status and HbA1c Evaluation

The diagnosis of DM was obtained from patients’ earlier medical records or determined prior to the cancer-related first-line treatment by DM specialists. Patients were categorized into the DM group and non-DM group according to the pre-existing condition. HbA1c was measured in all patients with DM before the initiation of treatment.

### Statistical Analysis

Continuous variables were compared using the t test or Kruskal-Wallis H test, and categorical variables were compared using the Chi-squared test. Bonferroni correction was used for multiple comparisons. OS was estimated with the Kaplan–Meier method and compared between subgroups by log-rank test. Multivariable Cox proportional hazards regression analysis using the backward stepwise selection was employed to identify independent factors of survival. The hazard ratio (HR) and 95% confidence interval (CI) was reported. Restricted cubic spline (RCS) analysis with covariates adjusted in the Cox proportional hazard model was used to visualize the continuous relationship between HbA1c level and OS (28). P-values for non-linearity were estimated by the Wald test. The optimal cut-off point of HbA1c for OS was determined using X-tile software version 3.6.1 (Yale University School of Medicine, New Haven, CT, USA) using a minimum P value from the log-rank chi-square test (29). A two-sided P value < 0.05 was considered statistically significant. SPSS version 23.0 (IBM, Chicago, IL, USA) and R software version 3.5.1 (http://lib.stat.cmu.edu/R/CRAN/) were used for analysis.

## Results

### Patient Characteristics and OS Comparison According to the Diabetic Status

In 1279 cases of advanced stage NSCLC, 300 (23.5%) had pre-existing DM with a median HbA1c of 6.0% (range: 4.5-10.7%). Compared with non-DM patients, DM patients were more likely to have greater BMI and CVD. When DM patients were further categorized into three tertiles according to the HbA1c level (low: 4.5-5.7, middle: 5.8-6.1, high: 6.2-10.7), those in the high tertile tended to be elder, male, ever smoker, non-adenocarcinoma, with greater BMI, CVD and wildtype EGFR (**Table 1**).

**Table 1:** Baseline characteristics of 1279 advanced LC patients with and without DM.

Variable	All	Non-DM	DM	P value^*^	DM patients			P value^#^
					Low tertile (HbA1c: 4.5-5.7)	Middle tertile (HbA1c: 5.8-6.1)	High tertile (HbA1c: 6.2-10.7)	
No. of patients	1279	979	300	/	88	93	119	/
Age (mean± SD), y	58.89 ± 10.71	59.08 ± 10.64	58.25 ± 10.90	0.238	51.91 ± 11.52	58.99 ± 8.65^a^	62.36 ± 9.86^ab^	<0.001
Age				0.933				<0.001
≤65	880 (68.80%)	673 (68.74%)	207 (69.00%)		76 (86.36%)	68 (73.12%)	63 (52.94%)^ab^	
>65	399 (31.20%)	306 (31.26%)	93 (31.00%)		12 (13.64%)	25 (26.88%)	56 (47.06%)	
BMI at baseline, kg/m^2^	22.48 ± 3.03	22.36 ± 3.03	22.85 ± 3.00	0.016	21.82 ± 2.81	22.97 ± 3.08^a^	23.51 ± 2.89^a^	<0.001
BMI				0.006				<0.001
≤24	887 (69.35%)	698 (71.30%)	189 (63.00%)		69 (78.41%)	62 (66.67%)	58 (48.74%)^ab^	
>24	392 (30.65%)	281 (28.70%)	111 (37.00%)		19 (21.59%)	31 (33.33%)	61 (51.26%)	
Sex				0.400				<0.001
Male	760 (59.42%)	588 (60.06%)	172 (57.33%)		36 (40.91%)	50 (53.76%)	86 (72.27%)^ab^	
Female	519 (40.58%)	391 (39.94%)	128 (42.67%)		52 (59.09%)	43 (46.24%)	33 (27.73%)	
Smoking history				0.647				<0.001
Non-smoker	740 (57.86%)	563 (57.51%)	177 (59.00%)		66 (75.00%)	58 (62.37%)	53 (44.54%)^ab^	
Ever smoker	539 (42.14%)	416 (42.49%)	123 (41.00%)		22 (25.00%)	35 (37.63%)	66 (55.46%)	
CVD				<0.001				0.001
Without CVD	1032 (80.69%)	861 (87.95%)	171 (57.00%)		62 (70.45%)	55 (59.14%)	54 (45.38%)^a^	
With CVD	247 (19.31%)	118 (12.05%)	129 (43.00%)		26 (29.55%)	38 (40.86%)	65 (54.62%)	
Stage				0.628				0.432
IIIB	497 (38.86%)	384 (39.22%)	113 (37.67%)		38 (43.18%)	32 (34.41%)	43 (36.13%)	
IV	782 (61.14%)	595 (60.78%)	187 (62.33%)		50 (56.82%)	61 (65.59%)	76 (63.87%)	
EGFR				0.637				<0.001
Wildtype	620 (48.48%)	471 (48.11%)	149 (49.67%)		29 (32.95%)	43 (46.24%)	77 (64.71%)^ab^	
Mutated	659 (51.52%)	508 (51.89%)	151 (50.33%)		59 (67.05%)	50 (53.76%)	42 (35.29%)	
Histology				0.064				<0.001
Adenocarcinoma	976 (76.31%)	759 (77.53%)	217 (72.33%)		73 (82.95%)	74 (79.57%)	70 (58.82%)^ab^	
Non-Adenocarcinoma	303 (23.69%)	220 (22.47%)	83 (27.67%)		15 (17.05%)	19 (20.43%)	49 (41.18%)	
First-line treatment				0.005				0.007
EGFR-TKIs	496 (38.78%)	397 (40.55%)	99 (33.00%)		37 (42.05%)	35 (37.63%)	27 (22.69%)^a^	
Chemotherapy/chemoradiotherapy	769 (60.13%)	568 (58.02%)	201 (67.00%)		51 (57.95%)	58 (62.37%)	92 (77.31%)	
Others	14 (1.09%)	14 (1.43%)	0 (0)		/	/	/	
De novo advanced NSCLC				0.794				0.655
Yes	1094 (85.54%)	836 (85.39%)	258 (86.00%)		74 (84.09%)	79 (84.95%)	105 (88.24%)	
No	185 (14.46%)	143 (14.61%)	42 (14.00%)		14 (15.91%)	14 (15.05%)	14 (11.76%)	

DM, diabetes; CVD, cardiovascular disease; EGFR, epidermal growth factor receptor; TKI, tyrosine kinase inhibitors.

^*^compared between patients with and without DM.

^#^compared among DM patients categorized into low, middle, and high tertiles according to the HbA1c level.

a P<0.05 compared with the low tertile group.

b P<0.05 compared with the middle tertile group.

**Figure 1:**
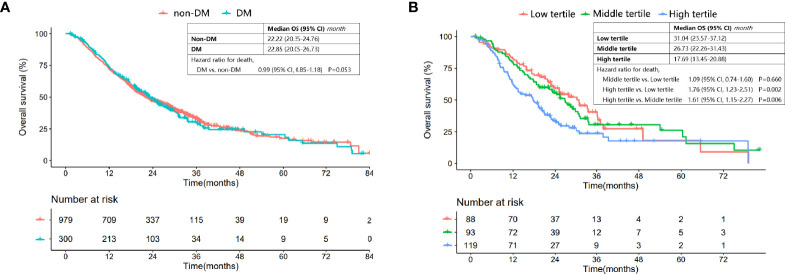
Comparison of OS in patients with and without DM, and DM patients categorized into different tertiles. **(A)** Comparison of OS between DM patients and nondiabetic patients. **(B)** Comparison of OS for DM patients in the low, middle and high tertiles.

The median follow-up of the entire cohort was 19.66 (Interquartile range: 11.01-27.98) months. The median interval between LC diagnosis and first-line treatment was 0.36 (interquartile range: 0.33-0.85) months. DM patients had a similar OS to nondiabetic patients [median (95% CI): 22.85 (20.05-26.73) *vs*. 22.22 (20.35-24.76) months, P=0.950] (**Figure 1A**). The multivariate Cox regression analysis also demonstrated that not DM status (HR: 0.929, 95% CI: 0.788-1.096, P=0.384) but EGFR wildtype (HR: 2.053, 95% CI: 1.650-2.555, P<0.001), smoking history (HR:1.404, 95% CI: 1.204-1.638, P<0.001), stage IV (HR:2.402, 95% CI: 2.058-2.802, P<0.001) and first-line EGFR-TKIs treatment (HR:0.732, 95% CI: 0.580-0.923, P=0.009) were independent prognostic factors for OS (**Supplementary Table 1**).

However, the Kaplan-Meier OS curve stratified by HbA1c tertile showed that DM patients in the high tertile had a significantly worse OS compared with those in the low and middle tertiles [median (95% CI) for the low, middle and high tertiles: 31.04 (23.57-37.12), 26.73 (22.26-31.43) and 17.69 (13.45-20.88), respectively; high *vs*. low tertile: P=0.002, high *vs*. middle tertile: P=0.006, middle *vs*. low tertile: P=0.660] (**Figure 1B**).

### Relationship Between HbA1c and OS

**Figure 2:**
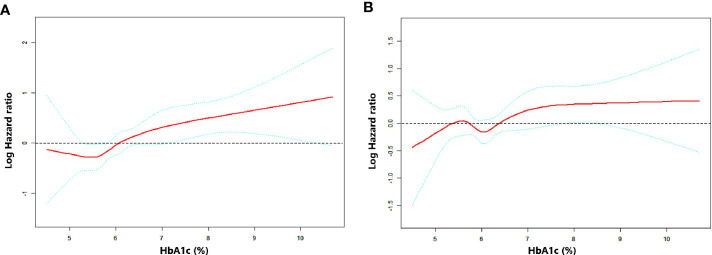
Association between HbA1c level and OS as plotted through unadjusted and adjusted restricted cubic splines model. **(A)** model 1 (unadjusted), **(B)** model 2 (adjusted by stage, EGFR status, smoking history and first-line treatment). The middle red line indicates the point estimates of Log hazard ratios and the blue lines indicate the lower and upper limits of the corresponding 95% confidence intervals. The horizontal broken line is at hazard ratio=1 (logHR=0). A significant non-linear association was observed **(A)** P for non-linearity=0.0096, **(B)** P for non-linearity <0.001).

RCS was performed to uncover the continuous relationship between HbA1c and OS. The effect of HbA1c on log-transformed HR for OS showed that there existed a non-linear but generally positive association between HbA1c level and OS, no matter unadjusted or adjusted by baseline characteristics (all P for non-linearity <0.01, **Figures 2A, B** and **Supplementary Figure 1**).

### Optimal HbA1c Cut-Off Determination

**Figure 3:**
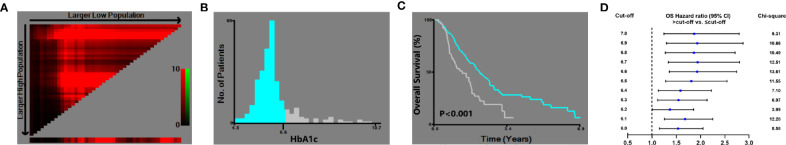
X-tile analysis of the optimal cut-off of HbA1c. **(A)** X-tile plots showed the chi-squared log-rank values created when the patients were divided into two groups. **(B)** The optimal cut-off point highlighted by the gray and blue panel. **(C)** The OS curves between the DM patients with HbA1c ≤6.6% and >6.6% (P<0.001). **(D)** Hazard ratio of OS with different HbA1c cut-off values in DM patients.

X-tile analysis showed that the optimal HbA1c cut-off value was 6.6 which showed the most significant prognostic difference on OS. DM patient with HbA1c ≤6.6% had a significantly longer OS compared with DM patients with HbA1c >6.6% (P<0.001) (**Figure 3**). The result remained significant in multivariate analysis after adjusting for baseline characteristics (DM with HbA1c>6.6 *vs*. DM with HbA1c ≤ 6.6: HR=1.593, 95% CI: 1.113-2.280, P=0.011) (**Table 2**).

**Table 2:** Univariate and multivariate Cox regression analysis of overall survival (n=300) in diabetic patients.

Characteristics	Univariate		Multivariate	
	HR (95%CI)	P value	HR (95%CI)	P value
DM with HbA1c>6.6 *vs*. DM with HbA1c ≤ 6.6	1.929 (1.355-2.746)	<0.001	1.593 (1.113-2.280)	0.011
Female *vs*. Male	0.624 (0.461-0.843)	0.002	1.111 (0.712-1.736)	0.642
Age>65 *vs*. ≤65	1.279 (0.943-1.735)	0.114	1.138 (0.832-1.558)	0.419
Ever smoker *vs*. Non-smoker	1.685 (1.263-2.248)	<0.001	1.392 (1.029-1.882)	0.032
With CVD *vs*. without CVD	1.339 (1.005-1.785)	0.046	0.895 (0.654-1.226)	0.490
BMI>24 *vs*. ≤24	1.203 (0.895-1.619)	0.221	1.053 (0.758-1.463)	0.757
Stage IV *vs*. IIIB	2.267 (1.654-3.108)	<0.001	2.674 (1.934-3.699)	<0.001
EGFR wildtype *vs*. EGFR-mutated	2.982 (2.202-4.038)	<0.001	2.970 (2.166-4.071)	<0.001
Non-adenocarcinoma *vs*. Adenocarcinoma	1.902 (1.403-2.578)	<0.001	1.278 (0.887-1.841)	0.188
First-line EGFR-TKIs *vs*. Non EGFR-TKIs	0.408 (0.289-0.576)	<0.001	0.925 (0.573-1.493)	0.750

DM, diabetes; CVD, cardiovascular disease; EGFR, epidermal growth factor receptor; TKI, tyrosine kinase inhibitors.

### Survival Benefit of Optimal Glycemic Control

**Figure 4:**
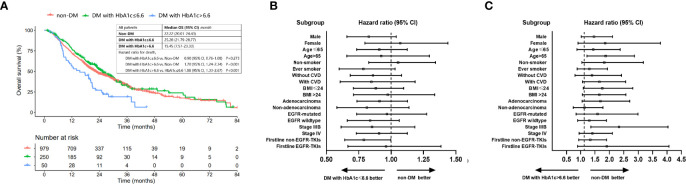
OS of patients with non-DM, DM with HbA1c ≤ 6.6% and DM with HbA1c>6.6%. **(A)** Comparison of OS among patients with non-DM, DM with HbA1c ≤ 6.6 and DM with HbA1c>6.6. **(B)** Forest plot for hazard ratio of OS for DM patients with HbA1c ≤ 6.6 *vs*. patients without DM. **(C)** Forest plot for hazard ratio of OS for DM patients with HbA1c>6.6 *vs*. patients without DM.

Well-controlled DM was determined as with HbA1c ≤ 6.6%. Survival benefit of optimal glycemic control was analyzed in all patients classified into three groups (non-DM, DM with HbA1c ≤ 6.6% and DM with HbA1c>6.6%). The median OS (95% CI) for nondiabetic patients, DM patients with HbA1c ≤6.6% and those with HbA1c > 6.6% was 22.22 (20.01-24.43), 25.28 (21.79-28.77) and 15.45 (7.57-23.33) months, respectively. Well-controlled DM (HbA1c ≤6.6%) patients had a comparable OS [HR (95% CI): 0.90 (0.76-1.08), P=0.273] compared to nondiabetic patients while patient with HbA1c>6.6% had a worse OS than patients without DM [HR (95% CI): 1.70 (1.24-2.34), P=0.001] and well-controlled DM patients [HR (95% CI): 1.88 (1.33-2.67), P<0.001] (**Figure 4A**). Subgroup analysis showed that the survival benefit of well-controlled DM with HbA1c ≤6.6% compared to non-DM patients was exhibited in all subgroups (**Figures 4B, C** and **Supplementary Figure 2**).

## Discussion

This retrospective study elucidated the impact of DM on the prognosis of advanced NSCLC. We found that not diabetic status but instead an elevated HbA1c level was linked with a worse prognosis in patients with advanced NSCLC. However, good glycemic control with HbA1c ≤6.6% could abolish the detrimental impact of DM on OS in advanced NSCLC. Survival benefit of proper glycemic control was unanimously prominent in all subgroups of advanced NSCLC.

Patients with coexisting lung cancer and DM is an unneglectable population and the prevalence of DM in NSCLC varied depending on ethnicity, population, staging, and histology. Approximately one out of four advanced NSCLC patients had preexisting DM in this study, which appears to be higher than previously reported prevalence of DM (11%-18.8%) in lung cancer (2, 3, 9, 30). A similar phenomenon of an increased prevalence of DM in a higher tumor stage was observed in the breast and colorectal cancer (31, 32). Findings that hyperglycemia and hyperinsulinemia associated with DM increase tumor cell proliferation and metastases also support the fact that DM tends to present with more advanced stage of NSCLC (33).

Patients with advanced NSCLC and DM have distinctive clinicopathological characteristics. Previous research on operable NSCLC showed that elderly patients, males, smokers, patients with cardiovascular comorbidities and squamous cell carcinomas had a greater chance of DM, which is consistent with our findings that DM patients with high HbA1c levels were more likely to be males, elder, smokers, having greater BMI, CVD, non-adenocarcinoma, and wildtype EGFR (34). Smoking status and BMI are well-known risk factors of DM (6, 35). Patients with DM tend to have an increased risk of CVD (36). Non-adenocarcinoma, mostly squamous cancer, is largely attributed to smoking and commonly presents in men. In addition, wildtype EGFR was more commonly seen in male, smoker and non-adenocarcinoma (37). Older patients with DM tended to receive less accurate glycemic control (38). All abovementioned intercorrelated features may hinder glycemic control in DM patients.

Contrary to previous research mostly focusing on the prognostic impact of DM itself, the current study placed more attention on the relationship between HbA1c level and survival. Our finding that not DM status but HbA1c level was related with increased mortality well explained why previous studies of mortality outcomes for NSCLC with pre-existing DM were conflicting (15, 16, 19, 20). We speculate that the ratio of good versus poor glycemic control is a critical factor in the analysis of survival. A recent retrospective study on postoperative NSCLC supported our result that the worst survival appeared in DM patients with a higher HbA1c (30). Accumulating data has deciphered the biological rationale underneath the association between HbA1c and outcome of cancer patients. Hyperglycemia as the critical feature of DM contributes to proliferation, apoptosis inhibition, metastasis, perineural invasion and resistance to cancer treatment (39, 40). In lung cancer, hyperglycemia facilitated metastasis by EMT induction and vascular destruction *via* oxidative stress and various mechanisms (39, 41). Hyperinsulinemia with increased levels of insulin-like growth factors also promotes tumor growth and causes EGFR-TKI resistance in NSCLC cells (33, 42).

There has been no standard of HbA1c threshold for advanced NSCLC with DM. For diabetic patients, HbA1c level is recommended to keep below 6.5-7% by the American Diabetes Association (43). However, individualization is proposed especially for those in the presence of other medical circumstances. In curative resected NSCLC, glycemic control with HbA1c <7% was proved to a positive prognostic factor (27). In the current study, the optimal HbA1c cut-off was determined by a more accurate and quantitative analysis which to our knowledge was the first application in advanced NSCLC patients with DM. We therefore recommend a target HbA1c level ≤6.6% for these patients based on the evidence of comparable OS between non-DM patients and DM patients with good HbA1c control.

EGFR-TKI has greatly improved the survival of advanced NSCLC with EGFR mutations. The current study unsurprisingly found that EGFR mutation status and first-line EGFR-TKIs therapy were independent prognostic factors for OS and EGRR-mutated patients had the longest and nearly three-year OS. It is noteworthy that glycemic control would be easier for patients eligible of EGFR-TKI therapy. Pretreatment of corticosteroids which are often contraindicated in DM due to the risk of disrupting glucose control is unnecessary for TKI therapy while corticosteroids are routinely included with chemotherapy (44). In addition, a possible synergistic effect of antidiabetic drug metformin with EGFR-TKIs should be considered (45–47).

There are several limitations in the study. First, confounding factors affecting the results may exist due to the retrospective nature. Increasing evidence demonstrated the prognostic role of concomitant medication including antibiotics, proton-pump inhibitor, corticosteroid and statins on the clinical outcome of advanced NSCLC treated with immune checkpoint inhibitors (48–50). Although our findings may not be applicable in the era of immunotherapy due to different treatment modalities in the last decade, metformin and corticosteroid may interfere with glucose control and have an influence on the prognosis (9, 46). In addition, genetic mutations except for EGFR were not collected due to incomplete information. Multicollinearity is also a noteworthy issue considering that DM patients with high HbA1c levels tended to be males, elders, smokers, obese, non-adenocarcinoma, with CVD and wildtype EGFR. We therefore assessed multicollinearity through variance inflation factor (VIF) and tolerance test. The VIF values were less than 2.5 and the tolerance values were between 0.1 and 1 for all covariates (data not shown) which indicated a relatively low degree of multicollinearity. However, a larger sample size may better eliminate the effect of multicollinearity. Second, impaired glycemic control might reflect underlying tumor characteristics such as tumor burden which in turn affect the prognosis. In addition, it may also modify the treatment strategies for example corticosteroid for brain metastases, which is worth further investigation. Third, previous study demonstrated an increased mortality at both high and low HbA1c levels for DM patients (51). In the current study, we assessed the optimal HbA1c upper limit but not the lower limit due to the relatively small sample size. Fourth, there is a possibility that DM may develop after cancer treatment which was not considered in this study. Fifth, we did not evaluate lung cancer-specific mortality due to the difficulty in classifying cause of death through telephone follow-up out of hospital. Further prospective large-scale study is warranted to confirm our findings.

In conclusion, impaired glycemic status negatively affected OS for patients with advanced NSCLC. Optimal glycemic control (HbA1c ≤6.6%) improved OS and is recommended in the management of advanced NSCLC with preexisting DM.

## Data Availability Statement

The raw data supporting the conclusions of this article will be made available by the authors, without undue reservation.

## Ethics Statement

The studies involving human participants were reviewed and approved by Shanghai Chest Hospital. The patients/participants provided their written informed consent to participate in this study.

## Author Contributions

JQ and WW conceived the study and wrote the manuscript. LW, JL, and LZ collected and analyzed the data. BZ, SW, WN, YZ, and YL contributed to the interpretation of the results and contributed to the final version of the manuscript. BH supervised the study. All authors contributed to the article and approved the submitted version.

## Funding

The study was funded by Shanghai Medical Research Program for The Outstanding Expert (No. TG20191101) and Shanghai Jiao Tong University School of Medicine PhD Student Innovation Fund (No. BXJ201843). The funders played no role in the study design, data collection and analysis, decision to publish, or preparation of the manuscript.

## Conflict of Interest

The authors declare that the research was conducted in the absence of any commercial or financial relationships that could be construed as a potential conflict of interest.

## Publisher’s Note

All claims expressed in this article are solely those of the authors and do not necessarily represent those of their affiliated organizations, or those of the publisher, the editors and the reviewers. Any product that may be evaluated in this article, or claim that may be made by its manufacturer, is not guaranteed or endorsed by the publisher.

## Supplementary Material

Supplementary Figure 1 Association between HbA1c level and OS as adjusted by age, gender, smoking history, CVD, BMI, stage, EGFR status, histology and first-line treatment (P for non-linearity <0.001).

Supplementary Figure 2 OS of patients with non-DM, DM with HbA1c ≤ 6.6% and DM with HbA1c>6.6% in subgroups.

Supplementary Table 1 Univariate and multivariate Cox regression of overall survival (n=1279) in the whole population. DM, diabetes; CVD, cardiovascular disease; EGFR, epidermal growth factor receptor; TKI, tyrosine kinase inhibitors.
