# Supplementary figures and images for: The Survival Benefit for Optimal Glycemic Control in Advanced Non-Small Cell Lung Cancer Patients With Preexisting Diabetes Mellitus
Source: Front Oncol. 2021 Nov 16;11:745150. doi: 10.3389/fonc.2021.745150 (PMC8635102; doi:10.3389/fonc.2021.745150)

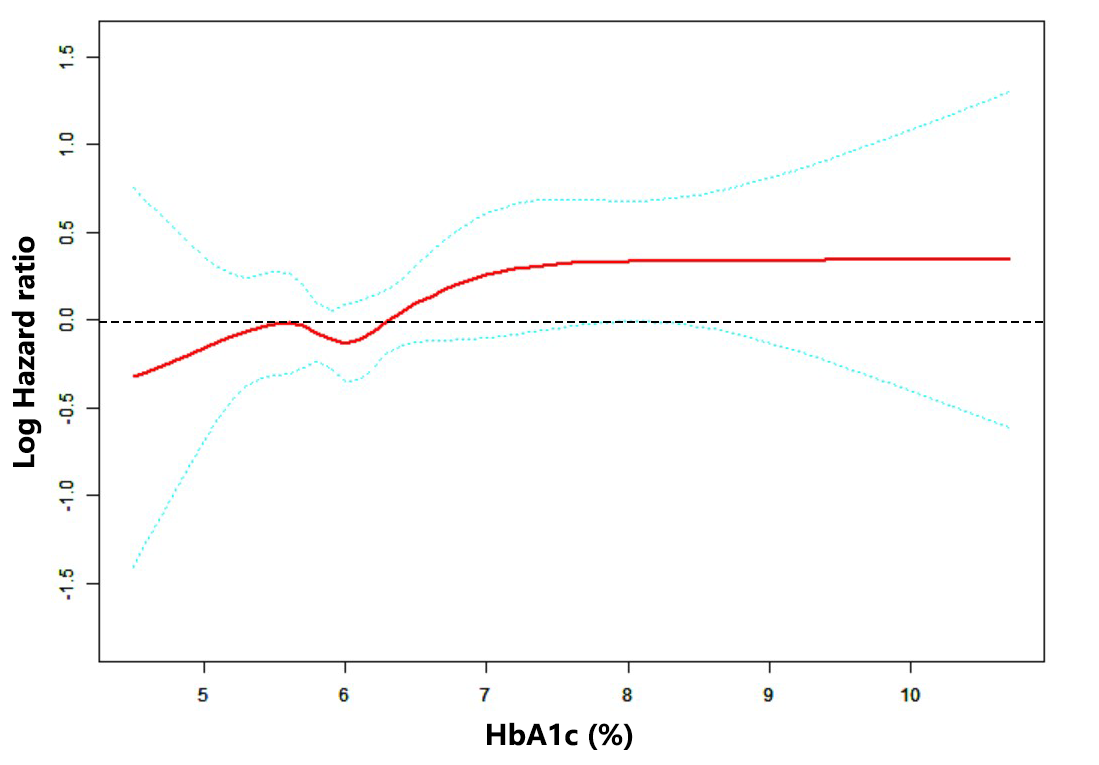

Supplement: Supplementary Figure 1 — Association between HbA1c level and OS as adjusted by age, gender, smoking history, CVD, BMI, stage, EGFR status, histology and first-line treatment (P for non-linearity <0.001). [file Image_1.tif]

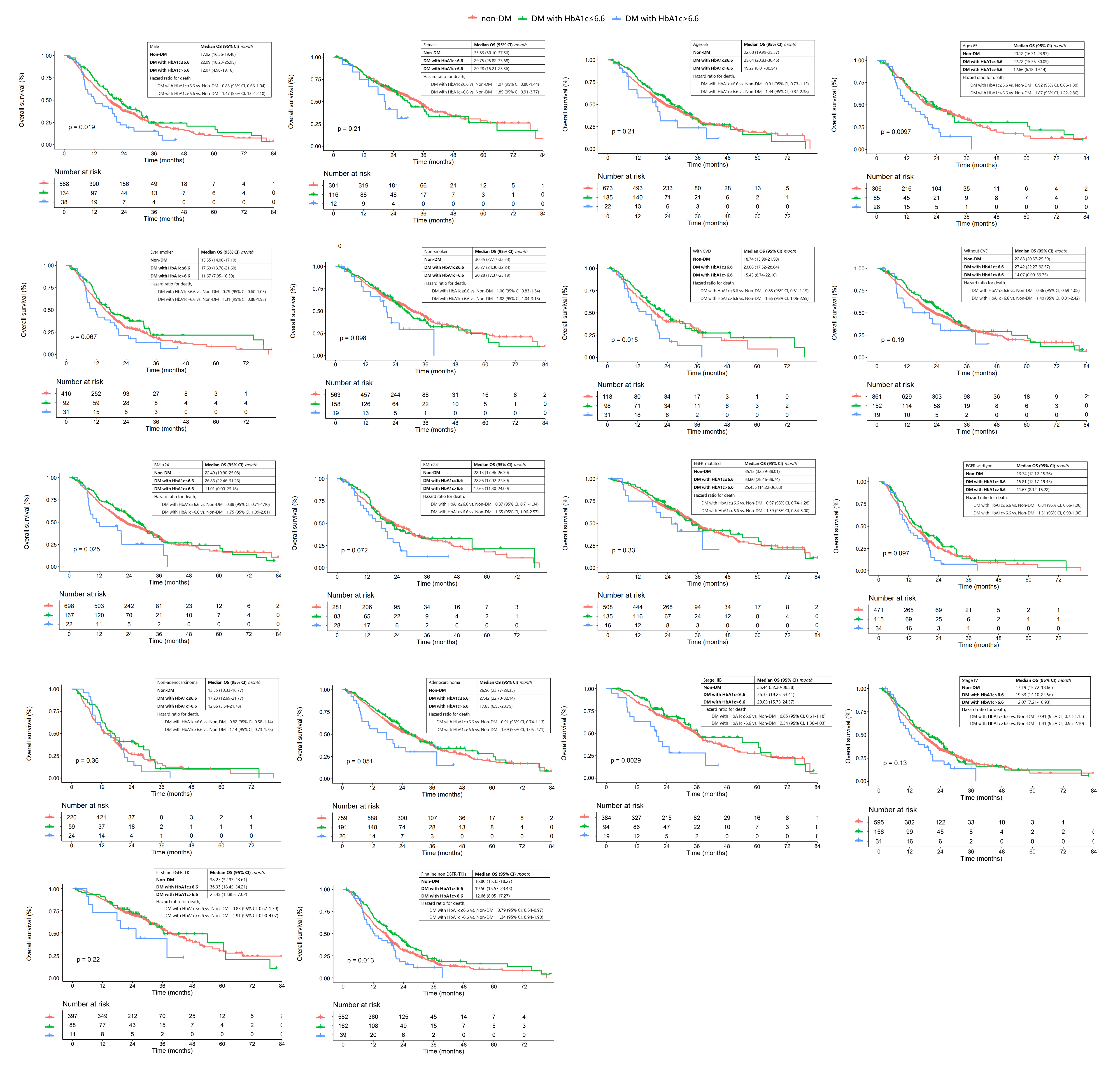

Supplement: Supplementary Figure 2 — OS of patients with non-DM, DM with HbA1c ≤ 6.6% and DM with HbA1c>6.6% in subgroups. [file Image_2.tif]
