# Supplementary material for: The Survival Benefit for Optimal Glycemic Control in Advanced Non-Small Cell Lung Cancer Patients With Preexisting Diabetes Mellitus
Source: Front Oncol. 2021 Nov 16;11:745150. doi: 10.3389/fonc.2021.745150 (PMC8635102; doi:10.3389/fonc.2021.745150)
Supplement: Supplementary Table 1 — Univariate and multivariate Cox regression of overall survival (n=1279) in the whole population. DM, diabetes; CVD, cardiovascular disease; EGFR, epidermal growth factor receptor; TKI, tyrosine kinase inhibitors. [file Table_1.docx]

**Supplementary Table 1. Univariate and multivariate Cox regression of overall survival (n=1279) in the whole population**

| Characteristics | Univariate | | Multivariate | |
| --- | --- | --- | --- | --- |
|  | HR (95%CI) | P　value | HR (95%CI) | P　value |
| With DM vs. Without DM | 1.005 (0.853-1.184) | 0.925 | 0.929 (0.788-1.096) | 0.384 |
| Female vs. Male | 0.542 (0.467-0.628) | <0.001 | 0.995 (0.806-1.229) | 0.966 |
| Age>65 vs. ≤65 | 1.097 (0.945-1.272) | 0.223 | 1.137 (0.978-1.322) | 0.094 |
| Ever smoker vs. Non-smoker | 2.016 (1.753-2.317) | <0.001 | 1.404 (1.204-1.638) | <0.001 |
| With CVD vs. without CVD | 1.221 (1.031-1.448) | 0.021 | 0.985 (0.817-1.199) | 0.878 |
| BMI>24 vs. ≤24 | 1.056 (0.909-1.226) | 0.476 | 1.040 (0.813-1.193) | 0.609 |
| Stage IV vs. IIIB | 2.316 (1.947-2.756) | <0.001 | 2.402 (2.058-2.802) | <0.001 |
| EGFR wildtype vs. EGFR-mutated | 3.111 (2.688-3.600) | <0.001 | 2.053 (1.650-2.555) | <0.001 |
| Non-adenocarcinoma vs. Adenocarcinoma | 0.542 (0.467-0.628) | <0.001 | 1.307 (1.101-1.552) | 0.002 |
| Firstline EGFR-TKIs vs. Non EGFR-TKIs | 0.356 (0.304-0.418) | <0.001 | 0.732 (0.580-0.923) | 0.009 |

DM: diabetes, CVD: cardiovascular disease, EGFR: epidermal growth factor receptor, , TKI: tyrosine kinase inhibitors
